# The Urosepsis—A Literature Review

**DOI:** 10.3390/medicina57090872

**Published:** 2021-08-25

**Authors:** Mădălin Guliciuc, Adrian Cornel Maier, Ioana Maria Maier, Alin Kraft, Roxana Ramona Cucuruzac, Monica Marinescu, Cristina Şerban, Laura Rebegea, Georgiana Bianca Constantin, Dorel Firescu

**Affiliations:** 1 Clinical Emergency County Hospital “Sf. Ap. Andrei”, 800578 Galați, Romania; guliciuc.madalin@gmail.com (M.G.); cristina.serban@ugal.ro (C.Ş.); laura_rebegea@yahoo.com (L.R.); dorelfirescu@yahoo.com (D.F.); 2 Faculty of Medicine and Pharmacy, Dunarea de Jos University, 800008 Galați, Romania; bianca.constantin@ugal.ro; 3 Emergency Military Hospital Galati, 800150 Galați, Romania; mioanamaier@gmail.com (I.M.M.); fixmonica@yahoo.com (M.M.); 4 Emergency Military Hospital Brașov, 500007 Brașov, Romania; alin.kraft@gmail.com; 5 University of Medicine and Pharmacy of Targu Mures, 540139 Târgu Mureș, Romania; roxana.cucuruzac@yahoo.com

**Keywords:** urosepsis, review

## Abstract

Urosepsis is a very serious condition with a high mortality rate. The immune response is in the center of pathophysiology. The therapeutic management of these patients includes surgical treatment of the source of infection, antibiotic therapy and life support. The management of this pathology is multidisciplinary and requires good collaboration between the urology, intensive care, imaging and laboratory medicine departments. An imbalance of pro and anti-inflammatory cytokines produced during sepsis plays an important role in pathogenesis. The study of cytokines in sepsis has important implications for understanding pathophysiology and for development of other therapeutic solutions. If not treated adequately, urosepsis may lead to serious septic complications and organ sequelae, even to a lethal outcome.

## 1. Introduction

The septic syndrome is the body’s abnormal systemic inflammatory response to an infectious process. It has an increased mortality rate and is the second leading cause of death in patients treated in intensive care units, after those with cardiovascular diseases [1,2].

Urosepsis represents a sepsis starting from the urinary tract or male genitals. Of the total cases of sepsis, urosepsis represents 9%–31%, the important percentage difference being dependent on the geographical location [3].

Given the high mortality rate, subsequent neurological and cognitive sequelae and the costs of increased hospitalization, patients with complicated urinary tract infections having a high risk of progression to urosepsis should be evaluated promptly and rigorously [4]. A retrospective study conducted over 10 years demonstrated that 78% of men and 54% of women with urosepsis associate urinary tract obstruction [5]. Moreover, superinfected urinary tract obstruction is mainly caused by reno-ureteral lithiasis (65%), followed by neoplastic pathology (21%), urinary tract abnormalities (5%) and postoperative complications (4%) [6].

Once the positive diagnosis and the favoring factors are established, the treatment can be initiated, which will include vital support, empirical antibiotic therapy respecting the principles of escalation and the surgical management of the associated urological pathology. From a surgical point of view, it is essential to practice early urinary drainage above the obstruction, or drainage and debridement of purulent collections [7,8].

The constant improvement of urosepsis management over time is demonstrated by increasing the incidence of sepsis with the decreasing overall mortality rate [9].

### 1.1. Etiology

The urinary tract infection can be clinically very varied, from asymptomatic bacteriuria to septic shock. The severity depends largely on the response of the host. It is important to remember that the disease can progress to an aggressive form in a short period of time [10].

Gram-negative bacteria are the main etiological factor of this pathology, as follows: *Escherichia coli* 50%, *Proteus* spp. 15%, *Enterobacter* and *Klebsiella* 15% and *Pseudomonas aeruginosa* 5%, while Gram-positive bacteria represent only 15% [11].

Data from the Pan European Prevalence (PEP) and Pan EuroAsian Prevalence (PEAP) studies reported a microbiologically proven infection in 74% of patients with urosepsis (uroculture 91%, blood culture 7%, other biological products 2%) [12].

*E. coli* strains isolated from patients with urosepsis have a lower prevalence of genetic traits phenotypically translated into virulence and are less likely to come from a uropathogenic clone than the strains isolated from patients with uncomplicated urinary tract infection. Polymicrobial bacteriuria is observed in elderly patients and in patients with chronic urological devices. Organisms isolated from patients with complicated urinary tract infection and urosepsis tend to be more resistant to antibiotics than strains isolated in uncomplicated urinary tract infection [13].

### 1.2. Pathophysiology

As the concept of host theory emerged, it was assumed that the clinical features of sepsis were the result of excessive inflammation [14]. However, it became apparent that infection triggers a much more complex, variable and prolonged immune response of the host in which both proinflammatory and anti-inflammatory mechanisms can contribute to infection elimination and tissue recovery and to the occurrence of acute organ damage and secondary infections [15]. In general, proinflammatory reactions, aimed at eliminating pathogens, are considered responsible for collateral tissue damage, while the anti-inflammatory response, important for limiting injury, is responsible for susceptibility to secondary infections, inflammatory process, by training and overcoming the body’s adaptation mechanisms, with the onset of shock, multiple organ dysfunction syndrome and death.

### 1.3. Innate Immunity

The first step in initiating the host’s response to the pathogen is the activation of innate immune cells, consisting mainly of macrophages, monocytes, neutrophils and natural killer (NK) T cells. The mechanism of neutrophils involve adhesion to the vascular endothelium, with associations of proteins (integrins and selectins). The integrins bind proteins on the surface of the endothelium and the selectins who are localized on the leukocytes and the endothelial cells facilitate an early adhesion of neutrophil and platelet [16,17].

The cytokine release in sepsis is the result of white blood cells, which also include macrophages. Normally the inflammatory response is balanced by an anti-inflammatory response with release the anti-inflammatory mediators (IL-4, IL-9, IL-10, epinephrine, transforming growth factor-β, soluble TNF-α receptors). This occurs by binding molecular structures associated with pathogens (PAMPs) such as bacterial endotoxins and fungal β-glucans to specific receptors on these cells resulting in activation of transduction pathways that cause transcription and secretion of proinflammatory cytokines, such as TNF-α, IL-1 and IL-6. In addition, some of the receptors, such as the NOD-like receptor group, can aggregate into larger protein complexes called inflammasomes that are involved in the production of crucial cytokines, such as IL-1β and IL-18, as well as caspases, which are involved in apoptosis. Proinflammatory cytokines cause leukocyte activation and proliferation, complement activation, overexpression of endothelial adhesion molecules, tissue factor production and induction of acute phase liver reactants [16,17] (Table 1).

### 1.4. Coagulation Disorders

The etiology of sepsis coagulation disorders is multifactorial. It is believed that hypercoagulability is determined by the release of tissue factor from destroyed endothelial cells but also from other sources: monocytes and polymorphonuclear cells. In patients with septic diseases the disseminated intravascular coagulation (DIC) with consumption of platelets and prolongation of clotting times is widespread. This condition leads to an altered hemostasis, which allows blood to clot and reduces blood flow. Tissue factor then causes systemic activation of the coagulation cascade, resulting in thrombin production, platelet activation and platelet-fibrin clot formation. These microthrombi can cause local hypoperfusion that leads to tissue hypoxia and organ dysfunction [17].

In patients with severe systemic inflammation there are low levels of plasma C protein, S protein and downward regulation of thrombomodulin secretion, which would normally temper the coagulation cascade, thereby allowing its activation. On the other hand, the patients with uncomplicated disease present a low-normal range of protein C concentrations. Plasminogen tissue activators are secreted in the endothelium as TNFα and IL-1β levels increase. These mediators may be triggered by induced expression of tissue factor (TF) on macrophages and endothelial cells. There is an imbalance between fibrin formation and fibrinolysis, increasing the amount of fibrin degradation products which contributes to the aggravation of microthrombosis [17,18] (Table 1).

### 1.5. Immunosuppression

The initial proinflammatory state is followed by a prolonged state of immunosuppression characterized by a low number of helper and natural killer T cells. Natural killer cells presented a 57% increasing in patients with sepsis vs. those with trauma. This is due to apoptosis but also to the decrease of inflammatory cytokines IL-6 and TNF, normally secreted by the stimulation of bacterial endotoxins This study revealed that apoptosis is present in only 4% in trauma patients compared with 12% in septic patients [19]. In septic patients, neutrophils were found to express fewer chemokine receptors and there was decreased chemotaxis in response to IL-8 [20]. Thus, the immune system is not able to organize an effective response to secondary bacterial, viral or fungal infections (Table 1).

### 1.6. Tissue and Organic Dysfunctions

The basic mechanism behind the dysfunction of tissues and organs in sepsis is the low intake and use of oxygen by cells due to hypoperfusion. The hypoperfusion caused by cardiovascular dysfunction is added to the coagulation disorders [21]. The incidence of septic cardiomyopathy varies from 18% to 60% in various studies. It is thought to be generated by circulating cytokines, such as TNF-α and IL-1β, which can alter myocyte contractility and mitochondrial function. Multiple studies have shown both systolic and diastolic dysfunctions, with decreasing beat volume and increasing telediastolic and telesystolic volumes [22,23].

Due to the arterial and venous dilation induced by inflammatory mediators and reduced venous return, hypotension and distributive shock occur. This is aggravated by the penetration of intravascular fluid into the interstitial space, due to the loss of the endothelial barrier function.

Hypoxia causes anaerobic glycolysis with the release of lactic acid and oxygen free radicals, which in turn will cause mitochondrial dysfunction, with decreased ATP, and these modifications due to major injuries in vital organs: lungs, kidneys, gastrointestinal tract or liver. Endothelial changes undermine the blood-brain barrier, causing the entry of toxins, inflammatory cells and cytokines into the central nervous system. They cause cerebral edema, synapse disruption and nerve tissue damage [24].

### 1.7. Clinical and Paraclinical Picture

According to the previous definition, urosepsis is a urinary tract infection proven by bacterial cultures, that associates the systemic inflammatory response syndrome (SIRS). The latter represents a continuous process consisting of four phases including localized infection in the urinary tract or male genitals that can progress to severe sepsis, multiple organ dysfunction and septic shock [7,8].

SIRS corresponds to the presence of 3 of the following criteria:

1. Fever, temperature above 38 °C, or hypothermia, below 36 °C;
2. Tachycardia—over 90/min;
3. Tachypnea—over 20 breaths/min or partial pressure of carbon dioxide in the arterial blood (PaCO_2_) < 32 mm Hg;
4. Leukocytosis > 12,000/mm^3^ or leukopenia < 4000/mm^3^, or the presence of immature cells in the periphery < 10%.

The updated definition states that urosepsis is a life-threatening organ dysfunction caused by the body’s abnormal response to a urinary tract infection [25]. The prolonged immune response of the host in which proinflammatory mechanisms should contribute to infection elimination is considered responsible for collateral tissue damage, while the anti-inflammatory response, important for limiting injury, is responsible for susceptibility to secondary infections. Thus, urosepsis with a high mortality rate requires a prompt therapeutic response. The body responds abnormally, so an immune cascade is involved in this process. According to the new definition, urosepsis also includes organ failure, so the old term for severe sepsis becomes irrelevant.

Septic shock is defined as a subgroup of sepsis in which abnormalities in cellular metabolism and circulatory disorders are significant enough to substantially increase mortality. Thus, a broader vision was expressed to differentiate septic shock from cardiovascular dysfunction associated with sepsis and to recognize the importance of abnormalities in cellular metabolism [25].

The multiple organ dysfunction syndrome (MODS) is characterized by dysfunction of two or more organs, requiring the intervention of the clinician to maintain homeostasis. To standardize the evaluation of these patients, the SOFA (sequential (sepsis-related) organ failure assessment) score [16] was developed, which evaluates the respiratory, nervous, and circulatory systems, the hepatic and renal functions and coagulation (Table 2). A SOFA score of 2 or higher have an overall mortality risk of approximately 10% [26].

The SOFA score is not intended to be used as a tool for patient management, but as a means of clinically characterizing a septic patient.

The utility of the score has previously been validated on large cohorts of critically ill patients [27,28]. The SOFA score demonstrated a satisfactory accuracy for predicting in-hospital mortality when applied to patients with sepsis with evidence of hypoperfusion at the time of presentation [29].

Given the fact that the SOFA score uses paraclinical data that require time and logistical resources, its use in screening patients with urinary tract infections is not recommended [26]. For the initial assessment of patients at risk of sepsis, the qSOFA score (quick SOFA) was developed, which incorporates cognitive impairment (Glasgow < 15), systolic blood pressure of 100 mm Hg or less and respiratory rate of 22/min or higher.

The criteria of QSOFA should be used to prompt clinicians to further investigate whether there is acute organ dysfunction, to initiate or intensify treatment and to increase the frequency of monitoring, or to consider transferring the patient to the intensive care unit. 

### 1.8. Biochemical Markers

Procalcitonin (PCT) is a peptide precursor of calcitonin. Serum levels of procalcitonin have been shown to increase dramatically in the response to bacterial infection [30]. Calcitonin synthesis is normally limited to thyroid C cells and, to a lesser extent, to other neuroendocrine cells [31]. Production is, however, activated in all parenchymal organs in response to bacterial infection, mediated by cytokines: interleukin-6 (IL-6), tumor necrosis factor-α (TNF-α) and interleukin-1β (IL-β). These tissues do not have the ability to cleave PCT to its mature form, calcitonin, leading to the accumulation of PCT [32]. In contrast, PCT production is attenuated by interferon-γ secreted primarily in response to viral infection [33]. This feature makes PCT a specific marker for bacterial infections. Procalcitonin compared to IL-2, IL-6, IL-8 and TNF-α also has the highest sensitivity and specificity for differentiating patients with SRIS from those with sepsis [34]. PCT becomes detectable within 2–4 h of the triggering event, reaching peak levels after 12–24 h. In the absence of a persistent PCT stimulus, it is eliminated, the half-life being 24–35 h. For this reason, the parameter can be useful for dynamic monitoring of patients [35]. Currently, the determination of procalcitonin is widely used by clinicians; however, there are a number of limitations generated mainly by the induction of procalcitonin expression by non-infectious diseases [36,37]. A procalcitonin/albumin ratio exceeding 0.44 is valuable for discriminating urosepsis from complicated urinary tract infection and may be superior to traditional biomarkers such as C-reactive protein and leukocyte count [38].

Mid-regional proadrenomedullin (MR-proADM) is a multi-tissue peptide to stabilize microcirculation and protect against endothelial permeability [39], both of which are recognized as having a significant role in the host’s pathophysiological response to sepsis. MR-proADM plays a decisive role both in inducing hyperdynamic circulation in the early stages of sepsis and in progression to septic shock [40]. MR-proADM differentiates sepsis from SIRS, with high specificity. Simultaneous determination of PCT and MR-proADM increases the sensitivity in the positive diagnosis of sepsis compared to the individual determination of each parameter [41].

Serum lactate is a marker of tissue hypoxia and is associated with mortality in sepsis [42]. Therefore, serum lactate should be monitored in patients with severe urinary tract infections.

### 1.9. The Cytokines—Biomarkers of Urosepsis

Although there are no ideal biomarkers available for sepsis, a number of existing biomarkers have been tested and have been shown to be useful in providing useful information to clinicians [43]. It has been reported that there is an association between plasma cytokine concentration, severity of sepsis and the risk of progression to multiple organ dysfunction [44].

Several studies have shown that elevated plasma levels of IL-18 have been correlated with unsatisfactory clinical outcome in patients with sepsis and therefore IL-18 may be an important biomarker in the evaluation of septic patients [45].

Midkine, also known as neuritis growth factor 2 (NEGF2), is significantly increased in patients with severe sepsis and septic shock. This increase is generated by generalized tissue hypoxia from sepsis. Midkine can be used in the differential diagnosis of SIRS versus sepsis and in the identification of patients with sepsis who are susceptible to cardiovascular failure and shock [46].

Leptin, a hormone produced by adipocytes, acts mainly in the hypothalamus to control body weight and energy consumption. Leptin is also involved in cell-mediated immunity and the expression of other cytokines. It has been established that there is a serum threshold of 38 mg/L of leptin useful in discriminating SIRS from sepsis [47].

IL-8 and monocyte chemotreating protein-1 (MCP-1) showed the best combination with SOFA in patients with sepsis on day 1. Elevated concentrations of IL-6, IL-8 and granulocyte colony stimulating factor (G-CSF) were correlated with the aggravation in the first 24 h of MODS or the lack of its improvement in the first 72 h. Several cytokines such as IL-1β, IL-4, IL-6, IL-8, MCP-1 and G-CSF were used to predict early mortality (<48 h), while IL-8 and MCP-1 were used in the 28-day mortality prediction. A multivariable analysis indicated that MCP-1 may be an independent indicator of the prognosis of sepsis [44].

These data, reinforced by subsequent meta-analyses, could lead to the development of a nomogram to characterize the severity of sepsis, the risk of progression to septic shock, MODS and death.

### 1.10. Treatment

The therapeutic management of these patients includes surgical treatment of the source of infection, antibiotic therapy and life support. For a more favorable result, in the case of patients at risk of progression to septic shock and organ dysfunction, a multidisciplinary approach is recommended. In patients with septic shock who were identified early and received intravenous antibiotic treatment and adequate electrolyte rebalancing, the hemodynamic management according to a strict EGDT protocol (early, goal-directed resuscitation) did not lead to an improvement in long-term mortality [48,49].

In addition, the costs of hospitalizing these patients were higher compared to those who were treated without using the EGDT protocol [50]. Patients hospitalized with urosepsis during the weekend have an increased mortality rate, which demonstrates the interdependence between the number of qualified staff compared to the number of patients. Each additional hour of medical care per day per patient was associated with a 2% decrease in the mortality rate [10].

The surgical treatment is an absolute emergency and involves the remediation of the bacterial source. This should be done promptly and minimally invasively in order not to add risks and surgical trauma to the clinical picture of urosepsis. Starting from this premise, the treatment of the obstructive cause is not desired, e.g., lithiasis, tumors, foreign bodies, congenital malformations, etc. This will avoid operative time and prolonged anesthesia, increased intracavitary pressure and secondary bacteremia, bleeding and possible intraoperative incidents.

Regarding the management of upper urinary tract obstruction, there is no rule in preferring external renal drainage (percutaneous nephrostomy) versus internal drainage (double autostatic ureteral stent J). The urologist decides between the two interventions depending on the nature, size and location of the obstacle, the degree of dilation of the renal collecting system, and the general condition of the patient [51,52,53].

In the case of patients with acute prostatitis and urinary retention, as well as of those with Fournier Syndrome (necrotizing fasciitis of the genitals), suprapubic urinary drainage will be performed (minimal cystostomy), thus avoiding the manipulation of the urethra. Of course, patients with Fournier Syndrome should benefit as soon as possible from the incision of the purulent collections, debridement and necrectomy. Given the involvement of anaerobic bacteria in the pathophysiological process of necrotizing fasciitis, no primary suturing will be performed [54].

Antibiotic therapy should be initiated promptly, less than one hour after the patient’s admission [55], but not earlier than the collection of biological samples for bacterial cultures, thus avoiding false negative microbiological results. The principle of escalation must be observed, using broad-spectrum antibiotics administered parenterally, in high doses, but adjusted according to creatinine clearance [56]. Once the antibiogram is available, treatment should be reconsidered based on bacterial resistance.

Life support is most often led by the intensive care physician and involves hydroelectrolytic rebalancing with crystalloid and albumin solutions. If these measures are not effective in achieving hemodynamic stability, vasopressors will be administered: first-line norepinephrine and dobutamine if there is myocardial dysfunction. If a SBP > 65 mmHg is not obtained, hydrocortisone can be administered. For the correction of severe anemia (hemoglobin < 8 mg/dL) isogroup blood products, iso-RH will be administered. For the prophylaxis of deep vein thrombosis, heparin with small molecular weight is administered. Proton pump inhibitors reduce the risk of stress ulcers.

Knowing the pathophysiology of septic shock, direct intervention was attempted to stop the cytokine storm. Corticosteroids suppress the production of cytokines, Annane et al. have shown that prolonged treatment with low doses of corticosteroids reduces long-term mortality in patients with septic shock [57]. Hemofiltration can remove some of the circulating cytokines; this translates clinically into improved blood pressure, respiratory function and improved SOFA score [58,59]. Ulinastatin, a human protease inhibitor and androstenediol, a metabolite of dihydroxystheniandrosterone, reduce plasma levels of TNF-α and IL-6 in rats with sepsis [60,61]. Experimental data show that biological neutralization of IL-18 may be a promising therapeutic approach in the treatment of sepsis; however, further studies will be needed to assess their full potential in the treatment of sepsis in humans [62]. GM-CSF treatment has improved clinical parameters in patients with sepsis and immunosuppression associated with sepsis, but did not improve the survival rate [63,64]. IL-10 neutralizing antibodies were tested to reduce immunosuppression in sepsis. Following this treatment, they have been shown to decrease mortality (animal model) due to increased IL-18 expression on the surface of NK cells and the IFN-γ response [65].

## 2. Conclusions

Urosepsis is a severe urological condition with a significant mortality rate.

In the prevention of urosepsis, the prudent use of antibiotics in the usual practice to avoid bacterial multidrug resistance, postoperative antimicrobial prophylaxis, especially in the case of endourology and the adequate management of urological pathology associated with urinary tract dilations play an important role.

An imbalance of pro and anti-inflammatory cytokines produced during sepsis plays an important role in pathogenesis. The study of cytokines in sepsis has important implications in understanding the pathophysiology, and in future development of other therapeutic solutions.

An early diagnosis and an appropriate treatment can reduce the costs of hospitalization, morbidity and mortality.

The management of this pathology is multidisciplinary and requires good collaboration between the departments of urology, intensive care, imaging and laboratory medicine. If not treated adequately, the urosepsis may lead to serious septic complications and organ sequelae, even to a lethal outcome.

## Figures and Tables

**Table 1:** Pathophysiology in urosepsis. Inflammatory mediators and their reactions.

Innate immunity	activation of macrophages, monocytes, neutrophils and NK T cells when binding with PAMPs	secretion of proinflammatory cytokines	leukocyte activation and proliferation, complement activation, overexpression of endothelial adhesion molecules, tissue factor production
		inflammasomes determine cytokines and caspase production	apoptosis
Coagulation disorders	destroyed endothelial cells, monocytes and polymorphonuclear cells release the tissue factor	thrombin production, platelet activation and platelet-fibrin clot formation	microthrombi cause local hypoperfusion that leads to tissue hypoxia and organ dysfunction
	increase levels of TNFα and IL-1β determine plasminogen tissue activators to be secreted in the endothelium	microthrombosis	
	low levels of plasma C protein, S protein and thrombomodulin	Activation of the coagulation cascade	
Immunosuppression	Apoptosis and decrease of inflammatory cytokines IL-6 and TNF	low number of helper and natural killer T cells	the immune system is not able to organize an effective response to secondary infection
	neutrophils express fewer chemokine receptors	decreased chemotaxis	

**Table 2:** SOFA score.

SOFA Score	0	1	2	3	4
**Respiratory system** The ratio between the partial pressure of arterial O_2_ and inspired O_2_ (PaO_2_/FiO_2_ mmHg)	>400	300–400	200–300	<200 and mechanical ventilation	<100 and mechanical ventilation
**Nervous system** Glasgow score	15	13–14	10–12	6–10	<6
**Cardiovascular system** Mean blood pressure (MBP) or administration of vasoactive agents	MBP > 70 mmGg	MBP < 70 mmHg	Dopamine < 5 μg/kg/min or dobutamine (regardless of the dose)	Dopamine > 5 μg/kg/min or adrenaline ≤ 0.1 μg/kg/min or noradrenaline ≤ 0.1 μg/kg/min	Dopamine > 15 μg/kg/min or adrenaline > 0.1 μg/kg/min or noradrenaline > 0.1 μg/kg/min
**Hepatic function** Bilirubin (mg/dL)	<1.2	1.2–1.9	2–5.9	6–11.9	>12
**Renal function** **Creatinine** (mg/dL)	<1.2	1.2–1.9	2–3.4	3.5–4.9	>5
**Coagulation** Thrombocytes × 10^3^/µL	>150	100–149	50–99	20–49	<20
